# Diabetes induces fibrotic changes in the lung through the activation of TGF-β signaling pathways

**DOI:** 10.1038/s41598-018-30449-y

**Published:** 2018-08-09

**Authors:** Girish Talakatta, Mohsen Sarikhani, Jaseer Muhamed, K. Dhanya, Bagganahalli S. Somashekar, Padukudru Anand Mahesh, Nagalingam Sundaresan, P. V. Ravindra

**Affiliations:** 1 Department of Biochemistry, CSIR-Central Food Technological Research Institute, KRS Road, Mysuru, 570020 India; 2 Department of Radiation Oncology, Houston Methodist Research Institute, Texas, 77030 USA; 3 Cardiovascular and Muscle Research Lab, Department of Microbiology and Cell Biology, Division of Biological Sciences, Indian Institute of Science, Bangaluru, 560012 India; 4 Department of Pulmonary Medicine, JSS Medical College, Jagadguru Sri Shivarathreeshwara University, Mysuru, 570015 India

## Abstract

In the long term, diabetes profoundly affects multiple organs, such as the kidney, heart, brain, liver, and eyes. The gradual loss of function in these vital organs contributes to mortality. Nonetheless, the effects of diabetes on the lung tissue are not well understood. Clinical and experimental data from our studies revealed that diabetes induces inflammatory and fibrotic changes in the lung. These changes were mediated by TGF-β-activated epithelial-to-mesenchymal transition (EMT) signaling pathways. Our studies also found that glucose restriction promoted mesenchymal-to-epithelial transition (MET) and substantially reversed inflammatory and fibrotic changes, suggesting that diabetes-induced EMT was mediated in part by the effects of hyperglycemia. Additionally, the persistent exposure of diabetic cells to high glucose concentrations (25 mM) promoted the upregulation of caveolin-1, N-cadherin, SIRT3, SIRT7 and lactate levels, suggesting that long-term diabetes may promote cell proliferation. Taken together, our results demonstrate for the first time that diabetes induces fibrotic changes in the lung via TGF-β1-activated EMT pathways and that elevated SMAD7 partially protects the lung during the initial stages of diabetes. These findings have implications for the management of patients with diabetes.

## Introduction

Diabetes causes profound long-term effects on multiple organs, such as the kidney, heart, skeletal muscle, brain, liver, and eyes. The gradual loss of function in these vital organs contributes to premature mortality in individuals with diabetes. At the tissue level, diabetes has been found to induce various pathological changes, including inflammation and fibrosis^1^. Tissue fibrosis initially results from tissue injury caused by pathological stimuli and is followed by the dysregulated production of extracellular matrix (ECM)^2,3^. A key cellular process that contributes to the development of tissue fibrosis is epithelial-to-mesenchymal transition (EMT). Although EMT is involved in physiological processes, such as embryogenesis and tissue repair, it can induce tissue fibrosis, which often represents the outcome of pathological chronic disease. In animal models, the inhibition of EMT has been demonstrated to be beneficial in attenuating the progression of tissue fibrosis, suggesting that EMT is an important process for ameliorating organ damage^4^. Diabetes can induce EMT through the sustained effects of hyperglycemia^5^. Further, diabetes-induced EMT is mediated primarily by the upregulation of TGF-β1, fibroblast-specific protein-1 (a key activator of EMT), and Snail (a transcriptional inducer of EMT) and the downregulation of nephrin, ZO-1, and P-cadherin^6–8^. The activation of TGF-β1 triggers the EMT program in epithelial cells, leading to the production of fibroblasts and the accumulation of ECM proteins in the tissue^4^.

Activated TGF-β1 forms a heteromeric complex with TGF-β receptors, leading to the activation of SMAD2 and SMAD3, which form a trimer with SMAD4. This complex translocates to the nucleus, where it activates the promoters of genes that encode EMT and ECM proteins and represses the expression of E-cadherin, an epithelial cell marker, thus promoting cell motility and invasion. In contrast, SMAD7 inhibits SMAD-dependent gene activation. TGF-β1 activation also results in the activation of SMAD-independent signaling components, such as Ras-ERK-MAP kinase, p38-MAP kinase and JNK, as well as the Rho GTPase and PI3 kinase/Akt signaling pathways. These pathways cooperate with TGF-β1/SMAD signaling to induce cellular responses that constitute TGF-β-induced EMT^9,10^. As a result of actin reorganization and the expression of EMT marker proteins, such as vimentin and fibronectin, epithelial cells acquire a mesenchymal phenotype. Furthermore, the increased expression and activity of matrix metalloproteases lead to ECM protein degradation and contribute to the invasive phenotype of mesenchymal cells^11^.

Although diabetes-induced complications have been demonstrated to affect multiple organs, the effects of diabetes on the lung are poorly characterized. A number of studies have found that individuals with either type 1 or type 2 diabetes present with pulmonary abnormalities, such as reduced forced vital capacity (FVC) and total lung capacity (TLC)^12,13^. Emerging evidence suggests that diabetes might affect the lung, in part through the induction of fibrotic changes in the tissue^14–17^; however, the effects of diabetes on the phenotype of alveolar epithelial cells (AECs) and on the involved cellular signaling pathways are unknown. Based on high-resolution computed tomography (HRCT) imaging and the evaluation of bronchoalveolar lavage fluid (BALF) samples from diabetes patients and a streptozotocin (STZ)-induced diabetic animal model, our findings provide scientific evidence that diabetes induces inflammatory and fibrotic changes in the lung. These changes are mediated by the induction of TGF-β1-mediated activation of both SMAD-dependent and SMAD-independent signaling pathways. Further, our results show that elevated levels of inhibitory SMAD7 contribute to the delayed response of the lung to the effects of diabetes.

## Results

### HRCT images and BALF from diabetic patients reveal fibrotic changes in the lung

**Figure 1:**
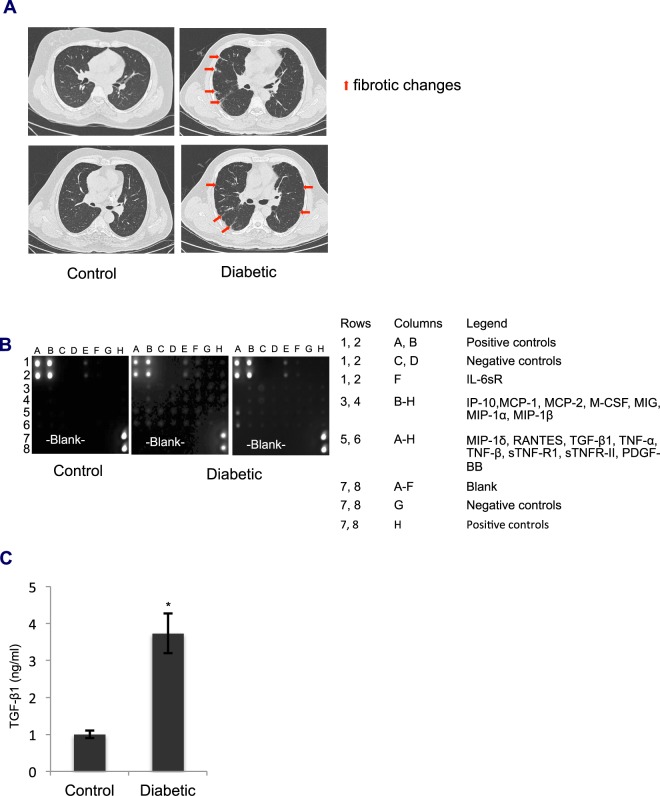
HRCT images, TGF-β1 levels and cytokine profiles in BALF from diabetic patients. (**A**) HRCT images of diabetic patients and normal controls. The presence of subpleural fibrotic strands at the indicated locations in the lung suggested that fibrotic changes were present in the lung, while most of the lung was similar to the control lungs. (**B**) Comparison of the human inflammation antibody array between diabetic patients (n = 4) and controls (n = 3). The intensity of dots corresponding to cytokines (IL-6sR (Rows 1, 2; Column F), IP-10, MCP-1, MCp-2, M-CSF, MIG, MIP-1α, MIP-1β (Rows 3, 4; Columns B-H), MIP-1δ, RANTES, TGF-β1, TNF-α, TNF-β, sTNF-RI, sTNF-RII, and PDGF-BB (Rows 5, 6; Columns A-H) was stronger in BALF from diabetic patients than in BALF from controls, suggesting that diabetes may induce inflammatory and fibrotic changes in the lung. (**C**) TGF-β1 levels in BALF from diabetic patients were estimated by using an ELISA kit. TGF-β1 levels were significantly higher (3.7-fold) in diabetic BALF than in control BALF. Bar graphs represent the mean ± standard error (SE); n = 3–4; *p < 0.05.

To explore the effects of diabetes on pathological changes in the lung, we first examined HRCT images of the lung from diabetic patients who were undergoing renal dialysis and had no history of chronic obstructive pulmonary disease (COPD) or other pulmonary diseases. The images revealed the presence of subpleural fibrotic strands at a few locations, which indicated fibrotic patches, while the majority of the lung parenchyma appeared normal (Fig. 1A). Fibrotic changes in the tissue are preceded by chronic inflammatory changes. To determine if inflammation provoked the formation of fibrotic strands in the lung, we examined the BALF from diabetic patients to determine the levels of inflammatory cytokines. We used a human inflammation antibody array (Abcam, Cambridge, UK), which revealed increases in the levels of MIP-1δ, IP-10, RANTES, TGF-β1, TNF-α, and MIP-1β in the diabetic BALF compared to the levels in the control samples. This finding indicated that diabetes might induce inflammatory and fibrotic changes in the lung (Fig. 1B). Since TGF-β1 is the master regulator of fibrotic pathways^11^, we next examined the levels of TGF-β1 in the BALF using an enzyme-linked immunosorbent assay (ELISA) kit (R&D Systems, Minneapolis, MN, USA). The TGF-β1 levels were significantly higher (by 3.7-fold) (Fig. 1C) in diabetic BALF compared to those in the controls, indicating that diabetes-induced pathological changes may involve the activation of TGF-β1 signaling pathways.

### Diabetes induces inflammatory and fibrotic changes in the lung

**Figure 2:**
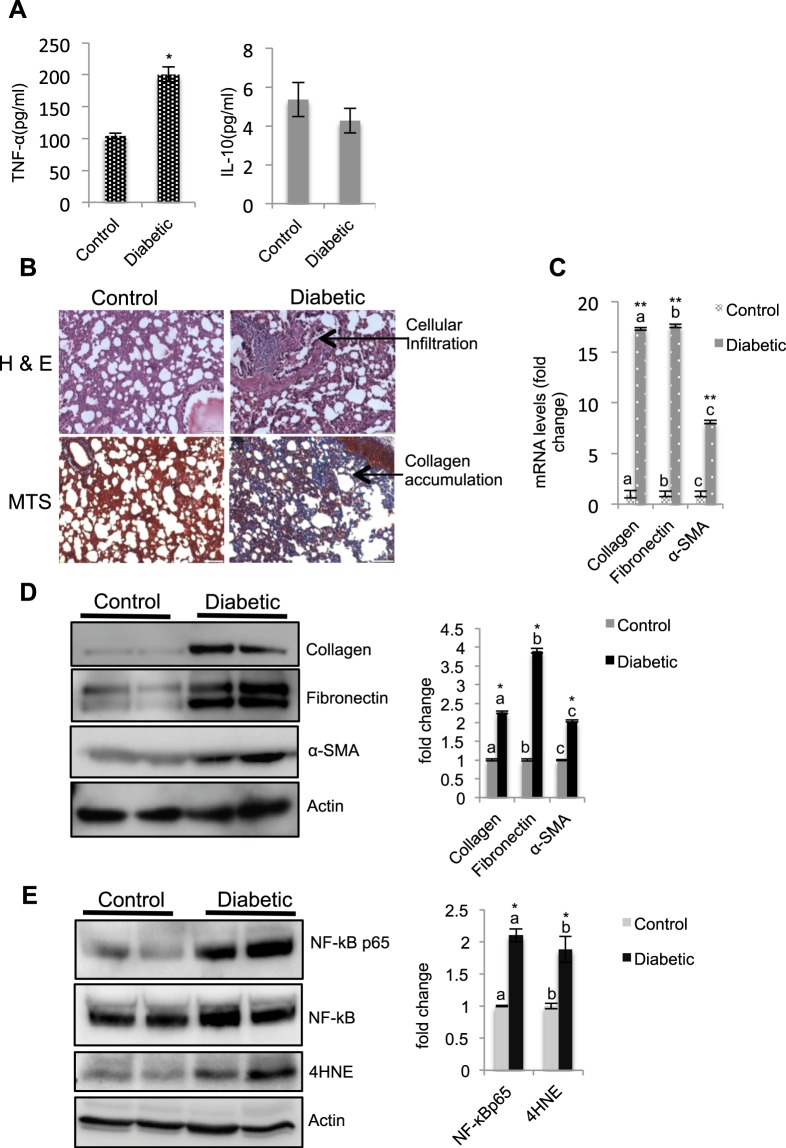
Diabetes induces inflammatory and fibrotic changes in the lung 12 wk after diabetes induction. (**A**) TNF-α and IL-10 levels in the serum were estimated by using an ELISA kit. TNF-α levels were significantly higher in diabetic animals than in control animals. Bar graphs represent the mean ± SE; n = 4; *p < 0.05. (**B**) H&E staining and MTS of lung tissue. The presence of intense cellular infiltration (upper right panel) and the appearance of blue-stained (lower right panel) regions in the diabetic tissue compared to the controls indicated inflammation and collagen accumulation, respectively. Scale bars represent 100 μm. (**C**) mRNA expression levels of collagen, fibronectin, and α-SMA in the lung by real-time PCR. Gene expression analysis was determined by the 2^−ΔΔCt^ method after normalization based on the CT values of 18 S rRNA and expressed as the fold change. Compared to the control lung, the diabetic lung showed a significant upregulation of collagen (17-fold), fibronectin (17-fold), and α-SMA (8-fold) levels. Bar graphs represent the mean ± SE; n = 6; (**) and the letters ‘a’, ‘b’, and ‘c’ on the bar graph indicate significant differences at p < 0.01. (**D**,**E**) Detection of collagen, fibronectin, α-SMA (upper panel), NF-κB, and 4HNE (lower panel) protein levels in the lung by western blot analysis. The diabetic lung showed significantly higher levels of collagen (2.3-fold), fibronectin (3.9-fold), α-SMA (2-fold), NF-κB (2-fold) and 4HNE (2-fold) than the control lung. Values represent the mean ± SE; (*) and the letters ‘a’, ‘b’, and ‘c’ on the bar graph indicate significant differences at p < 0.05; n = 3–6.

To understand diabetes-induced pathologies in the lung, we used the STZ-induced diabetic rat model. At 8 wk postinjection, the morphology of the lung appeared normal, but the animals had developed nephropathy and cataracts (data not shown). However, at 12 wk post-STZ injection, the lungs from diabetic rats appeared pale in color and had pinhead-sized nodules in certain regions of the lobes (Supplementary (S) Fig. 1A). Further, compared to the nondiabetic group, the diabetic group (SFig. 1B and C) showed significantly increased levels of TNF-α (2-fold) and decreased levels of IL-10 in the serum (Fig. 2A). Histopathological examination showed intense cellular infiltration in the interstitium in the diabetic group compared to the controls (Fig. 2B). The alveoli appeared to be shrunken, possibly due to the accumulation of collagen. Tissue sections stained with Masson’s trichrome stain (MTS) revealed the presence of blue-stained regions, confirming the accumulation of collagen (Fig. 2B, lower panel). The mRNA levels of collagen (17-fold), fibronectin (17-fold), and α-SMA (8-fold) were significantly higher in the diabetic tissue than in the control tissue (Fig. 2C). Western blot analysis showed significantly upregulated levels of collagen (2-fold), fibronectin (4-fold), and α-SMA (2-fold) in the diabetic tissue compared to the levels in the respective control tissues (Fig. 2D). Furthermore, the mRNA levels of both TNF-α (40-fold) and IL-6 (7-fold) were significantly higher in the diabetic tissue than in the control tissue (SFig. 2A). The inflammatory status was further verified by the upregulation of NF-κB (2-fold) and 4HNE levels (2-fold) (Fig. 2E). Additionally, the diabetic tissue showed significant increases in the levels of the Bax (32-fold) and Bcl2 (5-fold) genes and increases in caspase-3 compared to the levels in the control tissue (SFig. 2B,C). Taken together, the above results suggested that diabetes induces fibrotic and inflammatory changes in the lung.

### Diabetic cells exhibit a fibroblast phenotype and elevated expression levels of inflammatory and fibrotic markers

**Figure 3:**
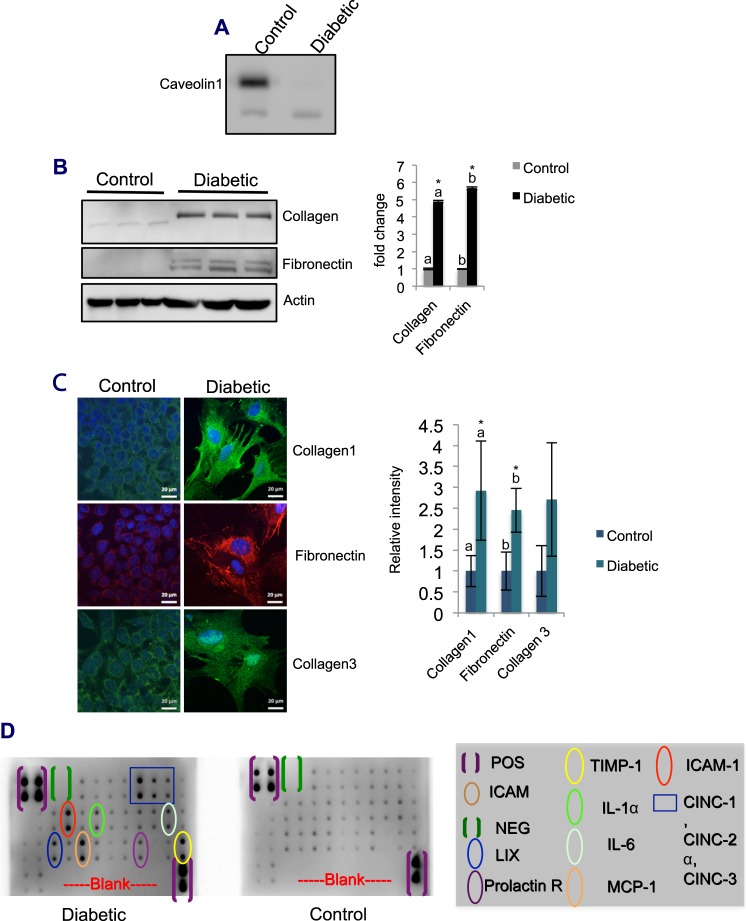
Expression levels of fibrosis and inflammatory markers in cultured cells isolated from lungs harvested from control and diabetic animals 12 wk after diabetes induction. (**A**) Caveolin-1 detection in cells cultured from the lung by western blot analysis. Caveolin-1 expression was detected in control cells; in contrast, in diabetic cells, caveolin-1 expression was not detected. The nonspecific band represents the loading control. (**B**) Expression levels of fibrosis marker proteins, such as collagen and fibronectin, in cultured cells by western blot analysis. Diabetic cells showed significantly higher expression levels of collagen (4.9-fold) and fibronectin (5.7-fold) than control cells. Values represent the mean ± SE; (*) and the letters ‘a’ and ‘b’ on the bar graph represent significant differences at p < 0.05; n = 3. (**C**) Detection of collagen 1, fibronectin, and collagen 3 expression in cells by the immunofluorescence method. The cells were cultured on imaging slides and incubated for 48 h. After incubation, the cells were fixed in 4% formaldehyde for 5 min. After permeabilization and subsequent blocking, the cells were incubated with a primary antibody overnight at 4 °C in a humidified chamber. After incubation with the appropriate secondary antibody and a subsequent phosphate-buffered saline (PBS) wash, the cells were counterstained with a nuclear stain. Images were acquired using a laser scanning confocal microscope (Zeiss, Germany). Images were analyzed by ZEN software (Zeiss, Germany). The intensity of fluorescence was higher in diabetic cells than in control cells, indicating elevated expression levels of collagen 1, fibronectin, and collagen 3. Scale bars are as indicated. Bar graphs represent the quantification (mean ± SE). (*) and the letters ‘a’ and ‘b’ indicate significant differences at p < 0.05; n = 3. (**D**) Detection of the expression levels of inflammatory cytokines in cultured cells by cytokine array. Lysates from control and diabetic cells were probed using a specific antibody-coated array according to the manufacturer’s instructions. The intensity of the dots in control and diabetic samples was recorded with the same exposure time. More intense dots were seen in the diabetic cytokine array than in the control cytokine array.

To determine if the above pathological changes in the tissue were reflected at the cellular level, cells were isolated from control and diabetic lungs by density gradient centrifugation. The detection of the epithelial cell marker caveolin-1^18^ in cells cultured from control lung tissue confirmed that a substantial proportion of the cells were AECs. On the other hand, caveolin-1 was not detected in cells cultured from the diabetic tissue, suggesting that those cells differed from the control cells (Fig. 3A).

Morphologically, diabetic cells were spindle-shaped and displayed a fibroblast phenotype, in contrast to the epithelial phenotype of the control cells. Further, the mRNA levels of collagen and fibronectin were substantially higher in diabetic cells than in control cells, confirming the fibroblast phenotype (SFig. 3A,B). Western blot analysis and immunofluorescence assays using anticollagen and antifibronectin antibodies detected significant increases in the levels of collagen (5-fold) and fibronectin (6-fold) in diabetic cells compared to the levels in control cells (Fig. 3B and C). Additionally, the mRNA levels of Bax, caspase-3, caspase-8, TNF-α, RANTES, IL-1, MCP-1, MIP-2, and iNOS were also significantly upregulated in these cells compared to the levels in the control cells. However, the mRNA levels of anti-inflammatory IL-10 in the diabetic cells (SFig. 3C) were slightly decreased compared to those in the control cells. The inflammation in diabetic cells was further confirmed using a cytokine array, which showed a substantial upregulation of the cytokines CINC-1, CINC-2α, CINC-3, ICAM1, MCP-1, LIX-1, TIMP-1, lL-1 and IL-6 compared to the levels in the control cells (Fig. 3D). Since the cells were cultured from the fibrotic portion of the lung, which resulted in a homogeneous population of fibroblasts, the levels of inflammation and fibrotic marker proteins detected in cultured cells were substantially higher than those detected at the tissue level. Taken together, these results suggested that the inflammatory and fibrotic changes in cultured cells were similar to the changes observed at the tissue level.

### Diabetes increases the expression levels of EMT markers in the lung

**Figure 4:**
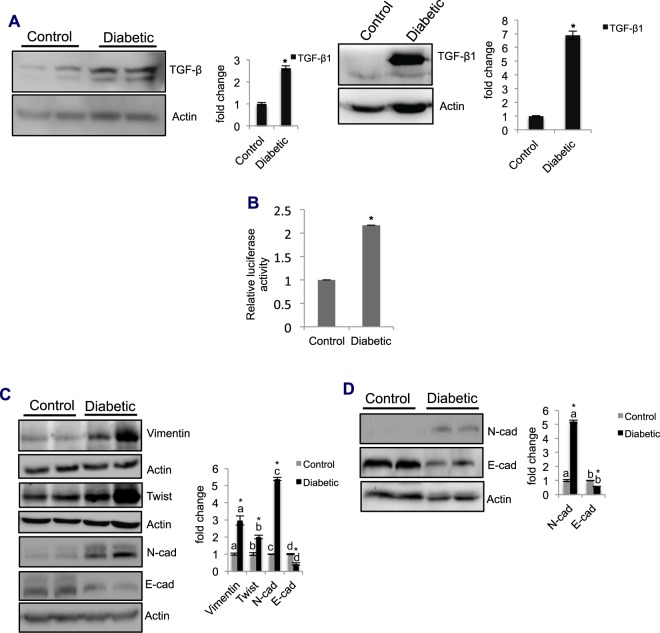
Diabetes induces EMT through TGF-β1 12 wk after diabetes induction. (A) Left panel: TGF-β1 detection in the lung by western blot analysis. Tissue lysates were probed for TGF-β1 levels by western blotting, and the levels were expressed as the fold change after normalizing to actin levels. TGF-β1 expression was significantly upregulated (2.6-fold) in diabetic tissue compared to the levels in control tissue. Bar graphs represent the mean ± SE; n = 3; *p < 0.05. Right panel: Detection of TGF-β1 in cells cultured from control and diabetic lungs. Cell lysates were probed for TGF-β1 expression by western blotting. Compared to the control cells, the diabetic cells showed increased levels of TGF-β1 (6.9-fold). In both tissue lysate and cell lysate, TGF-β1 was detected as two bands, with the upper band being more intense than the lower band. Values represent the mean ± SE; n = 3; *p < 0.05. (**B**) Detection of TGF-β1 promoter activity by luciferase assay. Cultured cells were cotransfected with a promoter reporter construct consisting of TGF-β1 promoter elements and a Renilla luciferase construct. At 24 h postincubation, the cells were harvested, and luciferase activity was determined using a luminometer. TGF-β1 promoter activity was expressed as relative luciferase units by normalizing the luciferase values to the Renilla luciferase units. TGF-β1 promoter activity was upregulated (2.2-fold) in diabetic cells compared to that in control cells. Values represent the mean ± SE; n = 3; *p < 0.05. (**C**) Detection of EMT marker protein levels in the lung by western blot analysis. EMT marker protein levels (vimentin, 3-fold; Twist, 2-fold; and N-cadherin, 5.4-fold) were significantly upregulated in the diabetic lung, while E-cadherin levels decreased (43%), compared to those in the respective controls. Values indicate the mean ± SE; n = 4; *p < 0.05. The letters ‘a’, ‘b’, ‘c’, and ‘d’ on the bar graph indicate significant differences. (**D**) Detection of EMT marker protein levels in cultured cells by western blot analysis. N-cadherin levels were significantly increased (5.1-fold), while E-cadherin levels were decreased (58%) in diabetic cells compared to those in control cells. Values indicate the mean ± SE; n = 4; *p < 0.05. The letters ‘a’ and ‘b’ on the bar graph indicate significant differences.

To elucidate the mechanisms of diabetes-induced fibrosis, we examined TGF-β1 levels in the diabetic tissue and in cells. TGF-β1 levels were significantly upregulated in the diabetic tissue (3-fold) and cells (7-fold) compared to those in the controls (Fig. 4A). TGF-β1 was detected as two bands, and the upper band was more intense than the lower band, indicating the involvement of posttranslational modifications. The levels of TGF-β1 were also verified at the mRNA level, which showed a significant upregulation (24-fold) compared to the levels in the controls (SFig. 4A). Furthermore, promoter assays revealed a significant increase (2-fold) in TGF-β1 promoter activity (Fig. 4B). Together, these findings confirmed that diabetes-induced fibrosis was partially mediated by the activation of TGF-β1. Furthermore, western blotting of diabetic tissue showed significant increases in vimentin (3-fold), twist (2-fold), and N-cadherin (5-fold) levels, with concomitant decreases in the levels of E-cadherin (by 57%), compared to the levels in the controls (Fig. 4C). The mRNA levels of EMT marker genes were also significantly upregulated in the diabetic tissue compared to those in the control tissue (SFig. 4B). Similar to our observations in tissues, compared to control cells, the diabetic cells also showed significant increases in the levels of N-cadherin (5-fold) and decreases in the levels of E-cadherin (approx. 52%) (Fig. 4D). Surprisingly, we noted a significant increase in the RNA levels of E-cadherin in the diabetic tissue compared to those in the control tissue (SFig. 4B). However, the cause of this increase in RNA levels was unclear. This increase could be due to the posttranscriptional regulation of E-cadherin. Taken together, these results suggested that diabetes induces pulmonary fibrosis, in part through the reactivation of the EMT pathway via the upregulation of TGF-β1.

### Diabetes-induced fibrosis involves the activation of TGF-β1-induced SMAD-dependent and SMAD-independent EMT pathways

**Figure 5:**
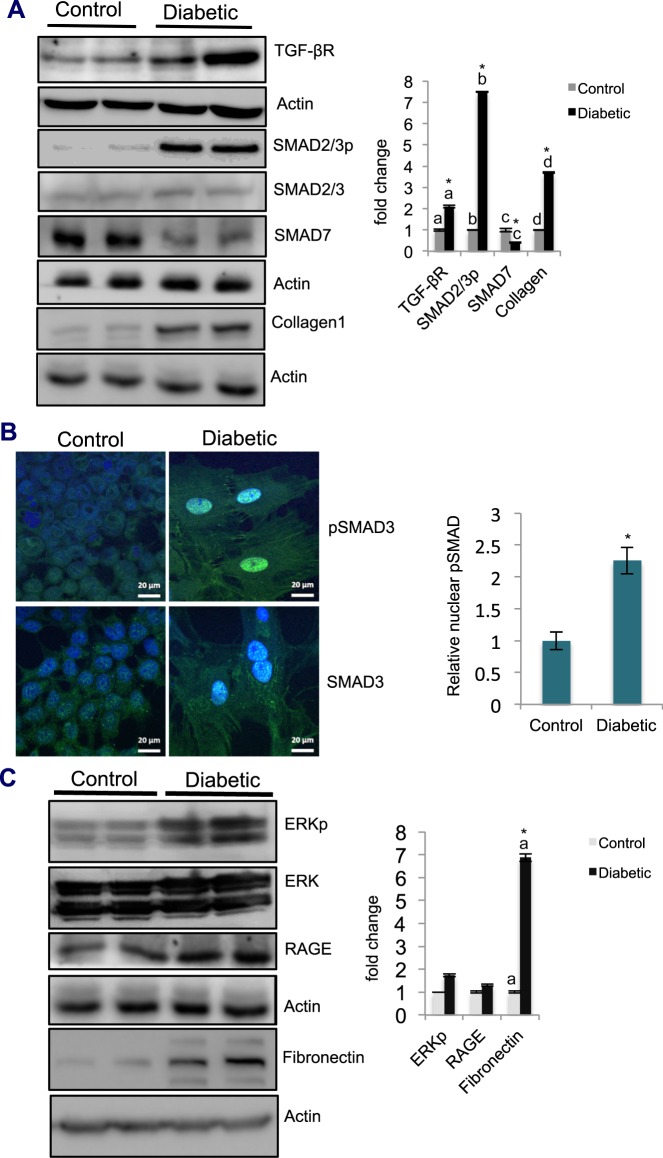
Diabetes-induced fibrosis involves TGF-β1-activated SMAD-dependent and SMAD-independent pathways. (**A**) Detection of proteins involved in the TGF-β1-activated SMAD-dependent signaling pathway in the lung by western blot analysis. Protein levels of SMAD-dependent pathway components (TGF-β1R, 2.1-fold; SMAD2/3p, 7.5-fold; and collagen, 3.7-fold) were significantly upregulated, while SMAD7 levels were significantly reduced (by 60%) in diabetic samples compared to those in the respective controls. Values indicate the mean ± SE; n = 4; *p < 0.05. The letters ‘a’, ‘b’, ‘c’, and ‘d’ on the bar graph indicate significant differences. (**B**) Localization of phosphorylated SMAD3 (SMAD3p) in cultured cells. Cells isolated from both control and diabetic lungs were cultured on imaging slides. At 48 h postculture, the cells were fixed in formaldehyde. After permeabilization using Triton X-100, the cells were blocked and incubated with fluorescein isothiocyanate (FITC)-conjugated anti-SMAD3p antibodies. Following staining with DAPI, the slides were examined for SMAD3p localization using a confocal microscope (Zeiss, Germany). Compared to the control cells, the diabetic cells showed enhanced localization of SMAD3p in the nucleus. Scale bars are as indicated in the figure. (*) indicates significant differences at p < 0.05; n = 4. (**C**) Detection of proteins involved in the TGF-β1-activated SMAD-independent signaling pathway in the lung 12 wk after diabetes induction by western blot analysis. Proteins of SMAD-independent pathways (ERKp, 1.7-fold; RAGE, 1.3-fold; and fibronectin, 6.9-fold) were upregulated in diabetic samples compared to those in control samples. Values denote the mean ± SE; n = 3; (*) and the letter ‘a’ on the bar graph indicate significant differences at p < 0.05.

To delineate the EMT pathways through which diabetes induces fibrosis, the levels of TGF-β1 receptor (TGF-β1R) and phospho-SMAD2/3 were examined. The results showed that the levels of TGF-β1R, SMAD2/3 activity, and the downstream target collagen were significantly elevated (by approx. 2-fold, 8-fold, and 4-fold, respectively), with a significant decrease in SMAD7 expression (60%), in the diabetic tissue compared to those in the controls (Fig. 5A). Furthermore, there was a significant increase in phospho-ERK levels (2-fold) and an increase in RAGE levels in diabetic tissue versus those in the control tissues (Fig. 5C). Confocal microscopy of diabetic cells revealed enhanced nuclear localization of phospho-SMAD3 (Fig. 5B). Together, these results suggested that diabetes-induced EMT, which leads to pulmonary fibrosis, was mediated by the activation TGF-β1-induced SMAD-dependent and SMAD-independent signaling pathways.

### SMAD7 levels are higher in the lung than in the kidney during the early stages of diabetes

**Figure 6:**
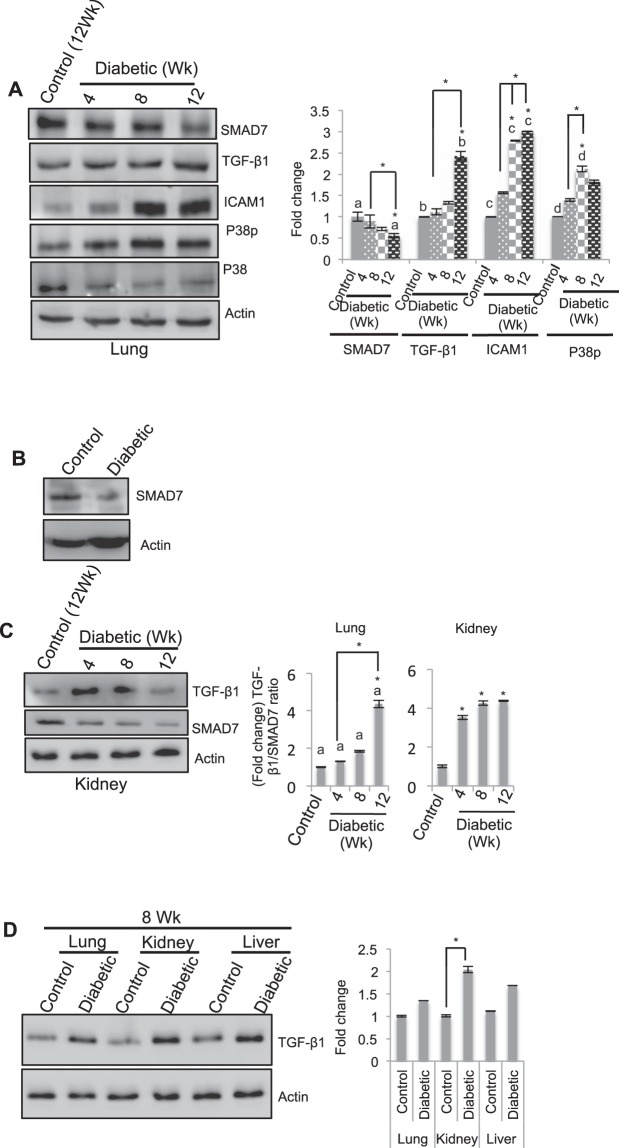
SMAD7 delays the effects of diabetes. (**A**) Detection of proteins involved in the TGF-β1-activated signaling pathway in the lung from 4 to 12 wk after diabetes induction by western blot analysis. SMAD7 levels in the diabetic lung were unchanged until 8 wk; however, these levels were downregulated significantly after 12 wk, with concomitant significant increases in TGF-β1, ICAM-1, and p38p. Values represent the mean ± SE; (*) and the letters ‘a’, ‘b’, ‘c’, and ‘d’ on the bar graph indicate significant differences at p < 0.05; n = 3. (**B**) Detection of SMAD7 in cultured cells. Cell lysates from control AECs and diabetic cells were probed using SMAD7-specific antibodies. Compared to control cells, diabetic cells exhibited decreased SMAD7 levels. (**C**) Comparison of the expression levels of TGF-β1 and SMAD7 in the lung (A) and the kidney from 4 to 12 wk after diabetes induction. The diabetic lungs showed no changes in the ratio of TGF-β1 to SMAD7 compared to the ratio in the control lungs until 8 wk. In contrast, the diabetic kidney showed a significant increase in this ratio at 4 wk compared to that in the control kidney, indicating that higher levels of SMAD7 and lower levels of TGF-β1 compared to the diabetic kidney contribute to the delayed response of the lung to the effects of diabetes. Values represent the mean ± SE; n = 3; (*) and the letter ‘a’ denote significant differences at p < 0.05. (**D**) Comparison of the expression levels of TGF-β1 among the lung, kidney and liver 8 wk after diabetes induction. The results showed that the kidney showed significantly higher expression levels than the other tissues, followed by the liver and the lung. This finding indicated that the kidney, followed by the liver, was more affected by TGF-β1-induced fibrosis than the lung. Values indicate the mean ± SE; n = 3; *p < 0.05.

The above results above indicated that inflammatory and fibrotic changes were visible in the lung 12 wk after diabetes induction. To investigate the possible reasons for the late onset of diabetes-induced pathological effects in the lung, we hypothesized that SMAD7, a negative regulator of TGF-β1 activation, protected the lung during the early stages of diabetes and that the levels of SMAD7 decrease with an increasing duration of diabetes. To test this hypothesis, we examined SMAD7 levels in the lung at 4, 8, and 12 wk after diabetes induction. We observed that there was no significant change in the level of SMAD7 compared to the level in the control until 8 wk after diabetes induction. However, the level of SMAD7 decreased significantly (45%) at 12 wk compared to the level in the control and compared to the level in the 4 wk diabetic control. Concurrently, the levels of TGF-β1 (2-fold), ICAM-1 (3-fold), and phospho-p38 (2-fold) were upregulated significantly compared to those in the respective controls and compared to those in the respective 4 wk diabetic controls (Fig. 6A). As anticipated, SMAD7 levels were substantially lower in the diabetic cells than in the control cells (Fig. 6B). These data suggested that the reduction in SMAD7 levels and the concurrent increase in TGF-β1 levels contributed to diabetes-induced EMT-mediated fibrosis.

We next compared the TGF-β1/SMAD7 ratio in lung and kidney tissues, which were harvested at the same time from the same animal. We observed that in the lung, the ratio was not significantly different from that of the control until 8 wk; however, the ratio increased significantly (by 4-fold) at 12 wk compared to the ratio in the control. On the other hand, the diabetic kidney showed a significant increase (3.5-fold) at 4 wk, with an additional increase (to 4.4-fold) 12 wk after diabetes induction, compared to the control (Fig. 6C). Furthermore, we observed that SMAD7 levels were comparatively higher in the lung than in the kidney at each time interval (Fig. 6C and SFig. 5). To determine if the delayed response of the lung was due to the presence of higher SMAD7 levels or lower TGF-β1 levels in the lung than in the kidney, we compared the TGF-β1 levels in the diabetic lung, kidney and liver with those in the respective controls after 8 wk of diabetes induction. We found that the diabetic kidney expressed significantly higher levels of TGF-β1, followed by the liver and the lung (Fig. 6D). These data suggested that TGF-β1-induced fibrosis affects the kidney more readily than the lung and the liver. Taken together, the above results suggested that the presence of higher levels of SMAD7 and lower levels of TGF-β1 in the diabetic lung than in the diabetic kidney contributes to the delayed response of the lung to the effects of diabetes compared to that of the kidney.

### Glucose restriction in diabetic cells activates mesenchymal-to-epithelial transition (MET), while prolonged exposure to high glucose induces the features of transformation

**Figure 7:**
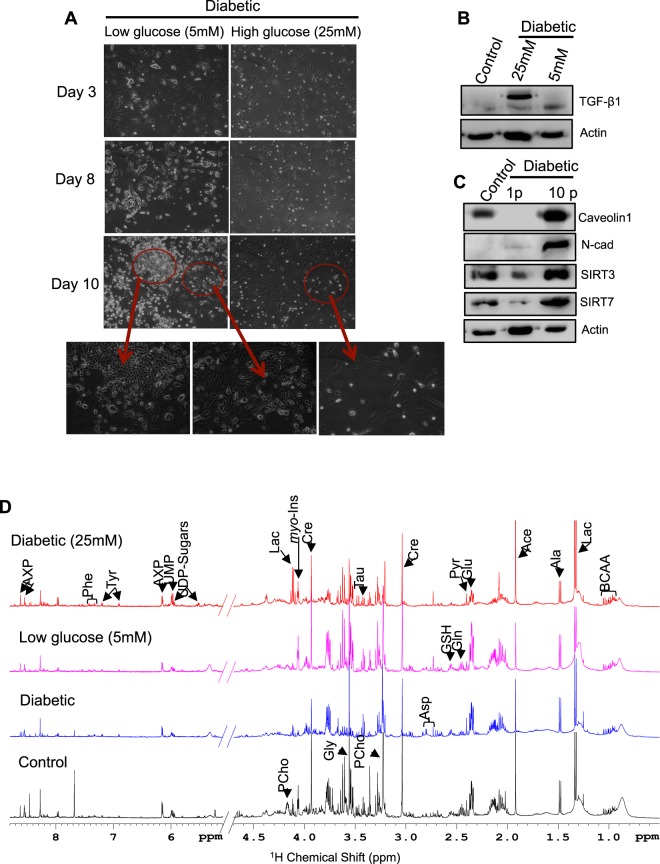
Glucose restriction promotes MET, while prolonged exposure induces features of transformation. (**A**) Morphological changes in diabetic cells cultured in medium with low and high glucose. Diabetic cells were cultured in the presence of low-glucose (5 mM) or high-glucose (25 mM) medium. At day 3 postculture, the cells grown in low-glucose medium began to show changes in morphology. At day 8 postculture, the cells cultured in low-glucose medium showed an epithelial morphology, while the cells cultured in high-glucose medium retained the mesenchymal cell morphology. At day 10 postculture, the cells in low-glucose medium showed patches of epithelial cells, while the cells cultured in high-glucose medium retained the mesenchymal phenotype. (**B**) Detection of TGF-β1 in diabetic cells cultured in low-glucose and high-glucose medium. The cells were lysed, and the lysates were probed using TGF-β1-specific antibodies by western blot analysis. An intense high-molecular-weight band was observed in diabetic cells cultured in high-glucose medium (25 mM) compared to that in cells grown in 5 mM glucose. Lysates from control AECs were used as negative controls. (**C**) Detection of marker proteins for cell proliferation by western blot analysis. Lysates from control AECs, diabetic cells, and diabetic cells cultured for 10p were probed using antibodies specific to the caveolin, SIRT3, and SIRT7 proteins. Compared to control cells, diabetic cells had reduced levels of the above proteins, but diabetic cells maintained in 25 mM glucose for 10p had elevated levels of the above proteins. (**D**) Metabolomics profiles of control cells, diabetic cells, and diabetic cells grown in medium with 5 mM or 25 mM glucose. The levels of *myo*-inositol, phosphocholine, creatine and AXP (AMP, ADP and AXP) were substantially decreased in diabetic cells compared to those in control cells. On the other hand, diabetic cells cultured in low-glucose (5 mM) medium exhibited a metabolomics profile similar to that of the control, corroborating the phenotypic protein changes observed in Fig. 7A,B. Furthermore, compared to control or glucose-restricted cells, diabetic cells that were maintained in high-glucose medium for an 10p exhibited substantial increases in lactate levels.

Diabetes-induced pathology is mediated by the durable effects of hyperglycemia^19^. In this study, diabetic cells were cultured in low- (5 mM) and high-glucose (25 mM) medium. At 3 and 8 days postculture, the cells grown in low-glucose medium showed an epithelial cell-like morphology. At 10 days postculture, small patches of epithelial cells were observed together with rare populations of fibroblasts. On the other hand, cells cultured in high-glucose medium retained the fibroblast morphology (Fig. 7A) even at 10 days postculture. These changes were subsequently verified at the RNA and protein levels. The protein levels of TGF-β1 were substantially decreased in cells cultured in low-glucose medium, while the TGF-β1 levels remained higher in cells cultured in high-glucose medium (Fig. 7B). Further, the expression levels of genes of the fibrotic and inflammatory pathways were substantially decreased in cells cultured in low-glucose medium compared to those of cells cultured in high-glucose medium (SFig. 6). These findings suggested that glucose restriction can promote MET and can help to overcome hyperglycemia-associated cell fibrosis. Additionally, these findings revealed that diabetes-induced EMT in the lung could be due to the prolonged effects of hyperglycemia.

To further explore the cellular changes in diabetic cells after 10 days of culture, the cells were grown for another 10 passages (10p) in high-glucose medium. The cells were harvested, and the levels of the cell proliferation marker proteins caveolin-1, N-cadherin^20^ and sirtuins (SIRTs) were examined^21,22^. As anticipated, the levels of caveolin-1, SIRT3 and SIRT7 were reduced under diabetic conditions compared to those observed under control conditions; however, N-cadherin levels were upregulated in fibroblasts. On the other hand, culturing diabetic cells in high-glucose medium for an additional 10p resulted in substantial increases in the levels of caveolin-1, N-cadherin, SIRT3 and SIRT7 compared to those in the control cells (Fig. 7C). To verify the above cellular changes, metabolomics profiling was conducted. As expected, the levels of *myo*-inositol, phosphocholine, creatine and AXP (AMP, ADP and AXP) were substantially decreased in diabetic cells compared to those in the controls. On the other hand, diabetic cells cultured in low-glucose medium exhibited a metabolomics profile similar to that of the controls; thus, these results were consistent with the phenotypic and protein changes in the cells, as observed in Fig. 7A,B. Furthermore, culturing the diabetic cells in high-glucose medium for an additional 10p resulted in substantial increases in the levels of lactate, which is a metabolite marker of cancer cells, compared to the levels observed under control or glucose-restricted conditions (Fig. 7D). Together, these results indicated that low glucose levels promoted MET in diabetic cells, while prolonged exposure to high glucose levels promoted the features of transformation.

## Discussion

Previous investigations suggested that the lung could be a target organ of diabetes based on clinical findings, such as reduced vital capacity and total lung capacity in diabetic patients^12,13^. However, there was no experimental evidence at the cellular level to elucidate the effect of diabetes on the lung. The presence of fibrotic strands, as detected in HRCT images, and elevated levels of TGF-β1 and other inflammatory cytokines in the BALF of diabetic patients (Fig. 1) with no history of COPD suggested that similar to other tissues, the lung could also be a direct target of diabetes. Further, the results indicated that cells in those fibrotic patches may contribute to the elevated levels of TGF-β1 and other inflammatory cytokines in the BALF. To elucidate the pathological effects of diabetes on the lung, we used the STZ-induced rat model. The infiltration of cells and the elevated levels of ECM proteins (Fig. 2) in the diabetic lung suggested that diabetes induces a direct effect on the lung, and this effect was mediated by the induction of inflammatory and fibrotic changes. A few other studies have also used STZ-injected rat models to study the impact of diabetes on fibrotic changes in the lung. One study observed patches of fibrotic abnormalities at 9 wk post-STZ injection^23^. In another study, diabetes-induced gene expression changes were observed after 4 wk of diabetes induction^24^. In our study, we also observed pulmonary fibrotic changes at 8 wk post-STZ injection, and those changes became prominent by 12 wk. Furthermore, we observed that this animal strain (strain IISc.) looked emaciated and died by 16–20 wk (4–5 months) after diabetes induction.

The elevated expression levels of EMT marker proteins in both diabetic cells and tissues demonstrated that diabetes-induced fibrotic changes were associated with the activation of EMT (Figs 3 and 4). Surprisingly, RNA levels of E-cadherin were elevated in diabetic tissue compared to those in control tissue. This finding indicated that diabetes-induced pathological effects are gradual and that not all of the tissue is affected at one time. This observation was similar to findings reported by Scharl *et al*.^25^, who noticed that E-cadherin levels were elevated with α-SMA during intestinal fibrosis. Since E-cadherin is a survival factor^26^, it is possible that the upregulation of E-cadherin might be a resistance mechanism used by cells during the transition from the epithelial phenotype to the mesenchymal phenotype. Furthermore, these findings also suggested the involvement of the posttranscriptional regulation of E-cadherin. However, these results require experimental evidence in the context of diabetes-induced EMT.

Our findings that showed that diabetes induced EMT (Fig. 4) were consistent with a study performed by Loeffler and Wolf.^27^, who showed that nephrons exposed to chronic hyperglycemia exhibited cellular changes consistent with EMT. In another study, diabetes-induced podocyte loss was observed due to EMT, which was mediated by TGF-β1 upregulation^28^. EMT is mediated by various signaling pathways. These pathways include SMAD-dependent and SMAD-independent pathways, which cooperate with TGF-β1 activation, thus resulting in EMT^9,10^. In our studies, upregulation of TGF-β1 receptor and phospho-SMAD2/3 levels, together with enhanced localization of phospho-SMAD3 in the diabetic lung and upregulated ERK and RAGE levels, suggested that diabetes-induced fibrosis was mediated by the activation of both SMAD-dependent and SMAD-independent pathways (Fig. 5). TGF-β1 pathways are negatively regulated by SMAD7 activation^29^. In our studies, decreased levels of SMAD7 in the diabetic lung compared to those in the control lung confirmed the presence of diabetes-induced TGF-β1 signaling in the lung.

The gradual increase in the levels of TGF-β1, ICAM-1, and phospho-p38, with a concurrent time-dependent decrease in SMAD7 levels, suggested that diabetes-induced fibrotic changes began at 8 wk postinduction (Fig. 6A,B). The time-dependent induction of effects of diabetes varies between organs. In other words, the time-dependent response to the effects of diabetes varies between organs. Substantial increases in the TGF-β1/SMAD7 ratio were observed in the kidney compared to that in the lung at 4 to 12 wk postinduction, suggesting that the response of the lung, unlike that of the kidney, was delayed and that the delay was due to the elevated levels of SMAD7 in the lung (Fig. 6C). This finding was further confirmed following the simultaneous comparison of TGF-β1 among the kidney, lung and liver, which showed that TGF-β1 levels were highest in the kidney, followed by the liver and the lung (Fig. 6D).

Previous studies have demonstrated that diabetes-induced pathological changes are mediated by the durable effects of hyperglycemia. A study by Yu *et al*.^5^ demonstrated that high glucose levels induce EMT in the peritoneal mesothelium by downregulating the expression levels of hepatocyte growth factor and bone morphogenic protein. In our study, following normal glucose culture conditions, the transition of the fibroblast phenotype of diabetic cells into the epithelial phenotype and the concurrent decrease in TGF-β1 levels indicated that fibrosis was mediated by chronic hyperglycemia. These findings also indicated that glucose restriction could promote MET (Fig. 7A,B). There are various types of pulmonary fibrosis, including idiopathic pulmonary fibrosis and familial pulmonary fibrosis. The presence of fibrotic changes in the lungs of diabetic patients who did not have prior pulmonary diseases indicated that the fibrotic changes induced in the lung were specific to diabetes and suggested a direct effect of diabetes.

**Figure 8:**
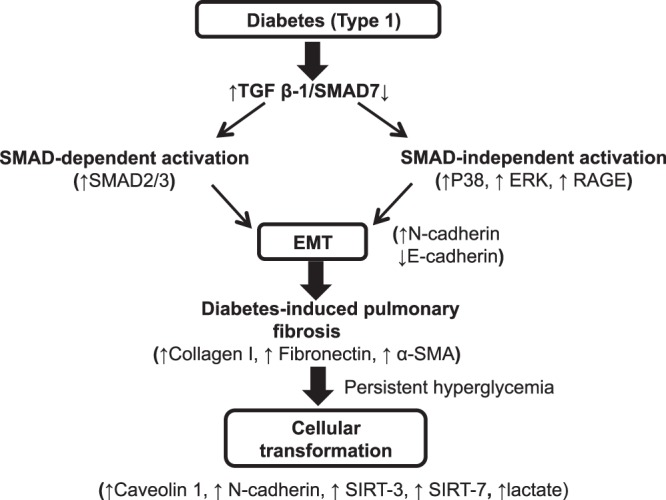
Summary of diabetes-induced pathological changes in the lung. Diabetes induces the upregulation of TGF-β1 and the downregulation of SMAD7, which subsequently activates both SMAD-dependent (elevated levels of SMAD2/3) and SMAD-independent signaling pathways (increased levels of p38, ERK, and RAGE), leading to EMT (decreased E-cadherin and increased N-cadherin levels) in the lung and the development of diabetes-induced pulmonary fibrosis (increased expression levels of collagen, fibronectin, and α-SMA). Persistent exposure of diabetic cells to high-glucose medium induces the expression of cell proliferation markers (increased levels of caveolin, N-cadherin, SIRT3, SIRT7 and lactate), which contributes to transformational changes in diabetic cells.

In certain cancerous cells, the expression levels of SIRT3 and SIRT7 were substantially higher than those in control cells, demonstrating that these proteins are cell proliferation markers^21,22^. Caveolin-1 and N-cadherin are upregulated in lung cancers^20^. The elevated levels of sirtuins, N-cadherin, and caveolin-1 (Fig. 7C), together with elevated levels of lactate (Fig. 7D), in cells cultured in high-glucose medium for a prolonged period suggested that chronic glucose induces the features of cellular transformation. Our observations are consistent with those reported in other studies^30,31^, which found that diabetes is an independent risk factor for the development of lung cancer. In a meta-analysis of observational studies, Lee *et al*.^31^ found that diabetes increases the risk of lung cancer, especially among female diabetic patients. In a similar study, Zhang *et al*.^32^ observed that treatment with metformin was associated with reduced risks of lung and respiratory cancer in diabetic patients. Similarly, a meta-analysis by Zhu *et al*.^33^ observed that metformin intake by diabetic patients reduces the risk of developing lung cancer. In addition, studies have found that metformin treatment increases the survival rate in patients with stage IV lung cancer^34^. In addition to lung cancer, diabetes has been found to be associated with the onset of ovarian cancer in female patients^35^ and with kidney cancer in both male and female patients^36^. Taken together, our findings that show transformational changes in diabetic cells after prolonged culture in high-glucose medium support previous studies. In summary, our findings demonstrate that diabetes induces pulmonary fibrosis. These changes were mediated by the activation of TGF-β1-induced EMT pathways. Decreased levels of SMAD7 in the diabetic lung promoted EMT. Additionally, glucose restriction in diabetic cells promoted MET, while prolonged culture in high-glucose medium induced the features of transformation (Fig. 8). A number of studies have found that microRNAs (miRNAs), including miR-182, miR-181a, and miR-375, promote the suppression of SMAD7 and thereby potentiate TGF-β1-induced effects in cancer cells^37–39^. Further, a study by Li *et al*.^40^ demonstrated that miR-5100 targets TOB2 to activate EMT in lung epithelial cells. In our laboratory, experiments are underway to investigate the role of miRNAs in diabetes-induced pulmonary fibrosis. Furthermore, similar to another study by Zheng *et al*.^41^, we also observed elevated TGF-β1 levels and pathological changes in the lung at 12 wk post-STZ injection. By this time, the diabetic animals had developed nephropathy and cataracts. Although few studies have reported an association between diabetic nephropathy and altered pulmonary function^42–44^, it remains to be investigated whether diabetic nephropathy or cataracts influence diabetes-induced molecular changes in the lung. Additionally, experiments are being conducted using SMAD7 knockout and transgenic models. Additional studies are required to determine if these fibrotic changes also occur in type 2 diabetes models.

## Materials and Methods

### Antibodies, kits, and cell culture reagents

Details of the antibodies, kits and cell culture reagents used in this study are listed in the Electronic Supplementary Information (ESM).

### Animal experiments

Animal experiments were carried out after obtaining approval from the animal ethical clearance committee (IAEC) of our institute (CSIR-CFTRI) (CFT/IAEC/37/2015). Animal handling and experimental methods were carried out according to the guidelines of the Committee for the Purpose of the Control and Supervision of Experiments on Animals (CPCSEA) from the Ministry of the Environment, Forest and Climate Change (Govt. of India, New Delhi, India). Wistar rats (male, strain IISc., India), weighing 150–160 g, were used in this study. A total of 60 animals, of which 45 animals were grouped for diabetes induction and 15 animals were grouped as controls, were used. A diabetic rat model was developed by intraperitoneal (I/P) injection with a single dose of STZ (Sigma, St. Louis, Missouri, USA) dissolved in sodium citrate buffer at 45 mg/kg body weight. Rats that received the same volume of sodium citrate buffer were used as controls. After 4, 8 or 12 wk of STZ injection, experimental animals and the respective controls were anesthetized using isoflurane and humanely sacrificed. Tissues were collected for histopathology, western blot analysis, RNA extraction and tissue culture studies.

### Isolation of AECs from the lung

Cells were extracted from the lung by the Percoll density gradient method, as described by Richards *et al*.^45^, with minor modifications (refer to the ESM for details).

### HRCT of the chest and collection and processing of BAL samples

HRCT imaging of the chest (ten patients) and BAL fluid collection (from four patients) procedures were performed in diabetic patients. These patients were aged greater than 50 years and had been diagnosed with diabetes in their late 40 s. These patients were on dialysis because of uncontrolled diabetes and had no history of smoking or COPD. Three age- and sex-matched healthy individuals served as a control group. The study was conducted after obtaining approval from the human ethical clearance committee (JSS University, Mysore, JSS/MC/IEC/810/2011–12) and after obtaining written informed consent from the patients and healthy individuals. All the procedures were carried out by following the “Ethical Guidelines for Biomedical Research on Human Participants” laid down by the Indian Council of Medical Research (ICMR) (Govt. of India, New Delhi, India). To collect the BALF, a sterile isotonic solution was infused through a bronchoscope wedged into the segmental bronchi, and the BALF was collected through gentle suction into a sterile container and examined for TGF-β1 and cytokine levels (refer to the ESM for details).

### Western blot analysis

Lysates were prepared by using radioimmunoprecipitation assay (RIPA) buffer (50 mM Tris-HCl, 1% NP-40, 0.5% sodium-deoxycholate, 1% sodium dodecyl sulfate (SDS), 300 mM NaCl, 2 mM ethylenediaminetetraacetic acid (EDTA) and 50 mM NaF) with a protease inhibitor cocktail (Sigma) on ice. A total of 60 μg of protein was resolved on an SDS-polyacrylamide gel (12%) and probed using antigen-specific antibodies (for details, refer to the ESM). Bands were detected by using a chemiluminescent substrate. Fold changes were calculated by NIH ImageJ software.

### Histopathology

Lung tissues were fixed in a 10% formalin solution and embedded in paraffin wax. Then, 5-μm-thick sections were stained with hematoxylin and eosin (H&E) or MTS (refer to the ESM).

### RNA extraction and quantitative real-time polymerase chain reaction (QRT-PCR)

The relative mRNA expressional levels were quantified by QRT-PCR (refer to the ESM for experimental details and primer lists).

### Immunofluorescence

Cultured cells were fixed with 4% formaldehyde and permeabilized with Triton X-100. After blocking with 5% bovine serum albumin (BSA), the cells were incubated with a primary antibody, followed by fluorescent Alexa Fluor 488-labeled (green) or Alexa Fluor 540-labeled (red) (Thermo scientific) secondary antibodies. After staining with a nuclear stain (4′6-diamidino-2-phenylindole (DAPI)), images were acquired using a laser scanning confocal microscope (Zeiss, Germany). Images were analyzed by ZEN software (refer to the ESM for details).

### TGF-β1 promoter activity

Cells were transfected with a TGF-β1 promoter reporter or the PGL3 basic (Promega, Madison, WI, USA) construct together with a Renilla luciferase plasmid using lipofectamine (Thermo Scientific) and incubated for 24 h at 37 °C (refer to the ESM for details).

### NMR-based metabolic profiling

The cells were harvested, aqueous metabolites were extracted, and NMR-based metabolic profiling was carried out as described previously (PMID: 22743333).

### Statistical analysis

Statistical differences between groups were determined with a two-tailed paired t-test and one-way ANOVA using Tukey’s multiple comparison test. *p* values less than 0.05 (*) and 0.01 (**) were considered significant.

## Electronic supplementary material

Supplementary Information
