# Supplementary material for: Diabetes induces fibrotic changes in the lung through the activation of TGF-β signaling pathways
Source: Sci Rep. 2018 Aug 9;8:11920. doi: 10.1038/s41598-018-30449-y (PMC6085305; doi:10.1038/s41598-018-30449-y)
Supplement: Supplementary file 1 — Supplementary Information [file 41598_2018_30449_MOESM1_ESM.pdf]

Diabetes induces fibrotic changes in the lung through the activation of TGF-β signaling pathways

Girish Talakatta*[2], Mohsen Sarikhani*[3], Jaseer Muhamed[3], K Dhanya[1], Bagganahalli S. Somashekar[1], Padukudru Anand. Mahesh[4], Nagalingam Sundaresan[3], P.V. Ravindra**[1]

1: Department of Biochemistry,
CSIR-Central Food Technological Research Institute, KRS Road, Mysuru, 570020.
2: Department of Radiation Oncology, Houston Methodist Research Institute, Texas, 77030, USA.
3: Cardiovascular and Muscle Research Lab, Department of Microbiology and Cell Biology, Division of Biological Sciences, Indian Institute of Science, Bangaluru, 560012.
4: Department of Pulmonary Medicine, JSS Medical College, Jagadguru Sri Shivarathreeshwara University, Mysuru, 570015.

*Contributed equally to this article
**Corresponding author: Email: raviravindra1@gmail.com, Ph: 91-8212514876

Materials and methods

1. Antibodies, kits, and cell culture reagents

Antibodies against the following proteins were used in our study: Caveolin1 (ab2910; 1:1000), TGF-β1 (ab92486; 1:1500), TGF-β1R (ab31013; 1:1000), RAGE (ab3611; 1: 1500), SMAD7 (Abcam; ab190987; 1: 2000), 4HNE (ab46545; 1: 2000), SMAD2/3 (ab202445; 1:1000); SMAD2/3p (ab63399; 1:1000), phospho-p38 (ab47363; 1:1500), p38 (ab32142; 1:1500) were purchased from AbCam (Abcam, Cambridge, UK). SMAD2/3p (CST- 9520; 1:1000), NF-κBp65 (CST-8242; 1:1000), caspase3 (CST-9662; 1:1000), N-cadherin (CST-4061; 1:2000); E-cadherin (CST-3195; 1: 2000), ICAM-1 (CST-4915; 1:1000), actin (CST-3700; 1:5000) were purchased from Cell Signaling (Cell Signaling Technology, Danvers, MA, USA). N-cadherin (Sigma; C3865; 1: 1000), Actin (A2228; 1:5000), vimentin (V6389; 1:1000), and twist (T6451; 1:2000) were purchased from Sigma (Sigma Aldrich St. Louis USA), SMAD2/3 (MAS-15663; 1; 500), α-SMA (MAS-11547; 1:5000), TGF-β1 (MA1-21479; 1:500) were purchased from Thermo Fisher Scientific (Thermo Fisher Scientific, Waltham, MA, USA), SMAD2/3 (SC-8332; 1:500), Collagen (SC-28888; 1:500; SC-8784; 1:1000), fibronectin (SC-9068; Sc-6952; 1:500), ERKp (SC-7383; 1: 2000), ERK (SC-93; 1:2000) were from Santa Cruz Biotechnology (Santa Cruz Biotechnology, Dallas, TX, USA). Human inflammation antibody array and rat inflammation antibody array kits were purchased from Abcam. TGF-β1 ELISA kit was purchased from RD Systems (R&D Systems, Minneapolis, MN, USA). TNF-α and IL-10 ELISA kits and SYBR green master mix were purchased from Sigma. The cDNA synthesis kit was purchased from Roche (Roche, Basel, Switzerland). Cell culture reagents, such as DMEM, were purchased from Gibco BRL (Thermo Fisher Scientific) trypsin-EDTA, FBS, antibiotic and antimycotic solutions were purchased from HI Media (HI Media, Mumbai, India); cell culture flasks and pipettes were purchased from

Eppendorf (Eppendorf, Hamburg, Germany). Rat lung alveolar epithelial cells were purchased from ATCC (ATCC, Manassas, VA, USA).

2. Animal experiments

Animal experiments were carried out after obtaining the approval from the institute's (CSIR-CFTRI) animal ethical clearance committee (IAEC) (CFT/IAEC/37/2015). Sixty (60) Wistar rats (male, strain IISc.,India), weighing 150-160g, were procured from the institute's animal house facility were housed in the same facility and were given water and food ad libitum. Forty-five (45) rats were intraperitoneally (I/P) injected with a single dose of STZ (Sigma) dissolved in sodium citrate buffer (0.1 M, pH 4.5) at 45mg/kg body weight. Fifteen (15) rats that received the same volume of sodium citrate buffer were used as controls. To circumvent the STZ-induced initial hypoglycemic mortality, animals were provided with 5% glucose solution in place of water for the first 48 h. Blood glucose levels were monitored using the AccuChek glucometer (Roche, Basel, Switzerland). Animals with fasting glucose levels >200 mg/dl were considered diabetic and were included in the study. Diabetic parameters, such as fasting blood glucose, urine volume and glycosuria, were determined in both control and diabetic rats. Additionally, inflammatory markers, such as TNF-α and IL-10, and insulin levels in serum were measured using the respective kits as per the manufacturer's instructions (Invitrogen, Carlsbad, CA, USA). Glycosuria was measured using 3,5-dinitrosalicylic acid methods. After 4, 8 or 12 Wk. of STZ-injection, experimental animals (n=10 each) and controls (n=3 each) were anesthetized using isoflurane and humanely sacrificed. Tissues were collected for histopathology, RNA extraction, western blot analysis and tissue culture studies.

3. Tissue processing and isolation of alveolar epithelial cells from lung tissue

Rats were anesthetized by isoflurane and dissected in the laminar hood; lung was collected for culture and for western blot, mRNA expression and histopathological experiments. For western blot analysis, tissues were stored at -80°C until use. For histopathological examination, tissues were collected in 10% formalin and stored at room temperature. Tissues were collected in RNAlater Tissue Storage Reagent (Sigma) and stored in -80°C for RNA extraction. Cells were extracted from the lung tissue by the Percoll density gradient method as described by Richards et al. (1987)[41] with minor modifications. Briefly, the lung tissue dissected from rat was transferred onto a petridish containing a 0.15 M NaCl solution with antibiotic and antimycotic solution reagent (1x). The tissue was minced in 0.25% trypsin solution, incubated for 10 min at 37°C and sterile filtered through a cell strainer (40μm, Corning Incorporated, Durham, NC, USA). The filtrate was subjected to Percoll density gradient centrifugation, in which 10mlof DMEM containing cells after centrifugation was layered on top of 10ml of each light and heavy Percoll gradient and was centrifuged at 1500 rpm for 10 min at room temperature. The cell pellet was resuspended in DMEM supplemented with 10% FBS, transferred to a T75 cm^2 cell culture flask and incubated at 37°C, 5% CO_2. Cells were observed until the monolayer was formed and were subsequently used for experiments.

4. Collection of BAL sample and its processing

Bronchoalveolar lavage (BAL) fluid was collected from four diabetic patients (male, aged above 50 years) with no history of COPD and who had previously developed nephropathy. Age and sex-matched three healthy individuals served as a control group. The study was conducted after obtaining the approval from the human ethical clearance committee (JSS University, Mysore, JSS/MC/IEC/810/2011-12) as well as written informed consent from the patients. All the procedures were carried out by following the

“Ethical Guidelines for Biomedical Research on Human Participants” laid down by the Indian Council of Medical Research (ICMR) (Govt. of India, New Delhi, India). Next, 150 to 200 ml of sterile isotonic solution was infused through the bronchoscope wedged into segmental bronchi and the samples were collected through gentle suction into a sterile container. Recovered BALF was stored at -80°C until use. To remove the mucus plugs, the BALF was filtered through a cell strainer (40μm) into a prechilled sterile 50ml centrifuge tube. Cells were pelleted by centrifugation at 1500 rpm for 10 min at 4°C. The cell pellet was stored at -80°C until use. The supernatant was concentrated by cryodesiccation and was subsequently reconstituted in RIPA lysis buffer with protease inhibitors. TGF-β1 levels and human cytokine array analysis was conducted using a TGF-β1 ELISA kit (R&D systems) and human inflammation array (Abcam), respectively, by following manufacturer’s instructions.

5. Western blot analysis

Tissue lysates were prepared by homogenization using RIPA buffer (50 mM Tris-HCL, 1% NP-40, 0.5% sodium-deoxycholate, 1% SDS, 300 mM NaCl, 2 mM EDTA and 50 mM NaF) with protease inhibitor cocktail (Sigma) on ice. Lysates were centrifuged, and the supernatant was collected and stored at -80°C until use. Protein concentration was determined by Lowry assay/BCA kit (Thermo Fisher Scientific). A total of 60μg of protein mixed with loading buffer was resolved on a SDS-polyacrylamide gel (12%). Proteins were transferred onto a polyvinylidene difluoride (PVDF) membrane. Membrane blocking was conducted at room temperature in 5% skim milk/BSA in TBST, followed by incubation with primary antibodies in 1% skim milk or 5% BSA overnight at 4°C on a rocker. The membrane was washed three times in TBST for 7 min each at room temperature. Subsequently, the membrane was probed with a horseradish peroxidase-conjugated anti-rabbit or anti-mouse secondary antibody in 1% skim milk for 1 h at room

temperature. After washing three times in TBST, the membrane was developed using ECL substrate (BioRad) and was scanned using the gel documentation system (Syngene, Cambridge, UK). The band intensity was calculated using ImageJ software (NIH, Bethesda, MD, USA).

6. Histopathology

Lung tissues were fixed in 10% formalin solution and embedded in paraffin wax. Next, 5-µm thick sections were cut using an automated microtome and were stained with hematoxylin and eosin (H&E) or Masson's trichrome stain. Slides were examined for morphological changes, and images were captured using an Olympus microscope (Model: BX-5, Japan; ProgRes C-5 software).

7. RNA extraction and Quantitative Real-Time PCR

Total RNA was extracted from cells or tissues using GenEx RNA extraction kit as per the manufacturer's protocol. DNA-free RNA was reverse-transcribed into cDNA using the Verso cDNA synthesis kit (Thermo Scientific). PCR was performed using the CFX96 Real Time PCR thermocycler (BioRad) and the SYBR Green method. The amplification of 18SrRNA was used for normalizing the expression levels of the target gene. Optimal primer concentrations and annealing temperature were determined by preliminary experiments. Gene expression analysis was determined by the $2^{-\Delta\Delta Ct}$method. The list of primers used is mentioned in the table 1 below.

8. Immunofluorescence

Cells were cultured on cell imaging slides (Eppendorf) until cells reached confluence. Cells were washed with PBS and were fixed with 4% formaldehyde for 5 min. Cells were permeabilized with Triton X-100 for 10 min. After blocking with 5% BSA in PBS for 1 h at

room temperature, cells were incubated with primary antibody (1:200 dilution in 1%BSA in PBS) against collagen 1, collagen 3, fibronectin, SMAD3p, or SMAD3 overnight at 4°C in a humidified chamber. After washing three times in PBS for 10 min each, cells were incubated with fluorescent Alexa fluor 488 (green) or 540 (red) (Thermo scientific) labeled secondary antibodies goat anti-mouse or anti-rabbit IgG secondary antibody (1:500 dilution, 1.5% BSA in PBS) for 1 h at room temperature. After washing with PBS three times for 10 min each, cells were counterstained with nuclear stain (DAPI- (4'6-diamidino-2-phenylidole). Images were acquired using a laser scanning confocal microscope (Zeiss, Germany). Images were analyzed by ZEN software.

9. TGF-β1 promoter activity

Transfection and reporter assays were performed in control and diabetic alveolar epithelial cells. Cells were transfected with TGF-β1 promoter reporter or PGL3 basic (Promega, Madison, WI, USA) construct together with a Renilla luciferase plasmid using lipofectamine (Thermo Scientific) and incubated for 24 h at 37°C. The Renilla luciferase activity was used for normalizing the transfection efficiency. At 24 h post-transfection, cells were lysed using the reporter lysis buffer (Promega). The supernatants were assayed for luciferase activity. The activation was determined based on the ratio between the normalized mean light units and that of the pGL3 basic construct control.

10. NMR-based metabolic profiling

Cells were grown as described above. Cells were harvested and aqueous metabolites were extracted, and NMR-based metabolic profiling was carried out as described previously (PMID: 22743333). Briefly, after cells reached 80-90% confluent, media was removed and cells were washed with phosphate buffer saline. Later, cells were trypsinized and cells were transferred into 2.5 ml vial. Cells were washed with 1 ml of

PBS for three times and 0.5 ml of cold methanol was added to the cell pellet. Cells were disrupted by ultrasonication, to this suspension 0.5ml of chloroform and 0.5ml of milliQ water was added, vortexed thoroughly and centrifuged for 30 min at 15000 rpm. Top aqueous layer was transferred in to clean 2.5 ml vial and dried using vacuum concentrator. Finally, the pellet was dissolved in 0.6 ml pH 7.4 phosphate buffer prepared D_2O and transferred into a 5 mm NMR tube. One-dimensional NMR experiment with water pre-saturation was acquired and chemical shift assignments of metabolites were carried out described previously (PMID: 22743333).

Table 1

LIST OF PRIMERS USED IN THE STUDY

Genes	Forward	Reverse
IL-1β	AGCTCCACGGGCAAGACATAGG	GGATGGCTTCCAAGCCCTTGAC
IL-6	CCGGAGAGGAGACTTCACAG	GGAAATTGGGGTAGGAAGGA
IL-10	GGCTCAGCACTGCTATGTTGCC	AGCATGTGGGTCTGGCTGACTG
iNOS	GTCACCTATCGCACCCGAGATG	GCCACTGACACTCCGCACAAAG
MCP-1	TCACGCTTCTGGGCCTGTTG	CAGCCGACTCATTGGGATCATC
MIP-2	GGCAAGGCTAACTGACCTGGAAAG	CACATCAGGTACGATCCAGGCTTC
Snail	AGTTGTCTACCGACCTTGCG	TGCAGCTCGCTATAGTTGGG
Slug	TCAGGAGCGTACAGCCCTAT	TACACGCCCCAAAGATGAGG
RANTES	ACTCCCTGCTGCTTTGCCTACC	TTGGCGGTTCCTTCGAGT
Bestrophin1	AAACCCTAACAGGACCCAAGC	AGGCACGAGAAGGAACCAAG
CTGF	GTGAGGAGTGGGTGTGTGAT	CAGTCGGTAGGCAGCTAGGG
E-cadherin	CCACCAGATGACGATACCCG	GAATCACTTCCGGTCTGGCA
N-cadherin	CCCAGGAAAAGTGGCAGGTA	GTCTCTCTTCTGCCTTTGTAGACG
MMP9	CAAGGACGGTCGGTATTGGA	CGTGCGGGCAATAAGAAAGG
MMP3	CCTCGTGGTACCCACCAAAT	AGAACAAGACTTCTCCCCGC
Col1a2	TAAAGAAGGCCCTGTGGGTCTC	GATGGCCTTTCTCACCAGGTT
α-SMA	CGGGCTTTGCTGGTGATG	GGTCAGGATCCCTCTCTTGCT
Fibronectin	GTGATCTACGAGGGACAGC	GCTGGTGGTGAAGTCAAAG
Bax	TGGCAGCTGACATGTTTGCT	TTTAGTGCACAGGGCCTTGAG
TGF-β	AGGACCTGGGTTGGAAGTGG	AGTTGGCATGGTAGCCCTTG
SMAD1	AGGAGAGGTGTATGCGGAGT	GGTGGTAGTTGCAGTTCCGA
Vimentin	CAGGATTTCTCTGCCTCTTCCA	CCTGTCCGTCTCTGGTTTCAAC
TNF-α	CATCCGTTCTCTACCCAGCC	AATTCTGAGCCCGGAGTTGG
Twist1	CCGGAGACCTAGATGTCATTGTT	CACGCCCTGATTCTTGTGAAA

SGK1	ACCTCCTCCAAGTCCCTCTC	TCTGCCTTGTGCCTTGCTA
Ctnna1	CGTTCGCCGCAGAAATGAC	ACCTGTGTAACAAGAGGCTCCAA
Caspase 3	TGTGAGGCGGTTGTAGAAGAGTTTC	GCACACCCACCGAAAACCAG
Caspase 8	GTGTGGGGTAATGACAATCTCG	CTCTTCAAAGGTCGTGGTCAAA
18S rRNA	GGACCAGAGCGAAAGCATTTGC	CGCCAGTCGGCATCGTTTATG

Supplementary figures

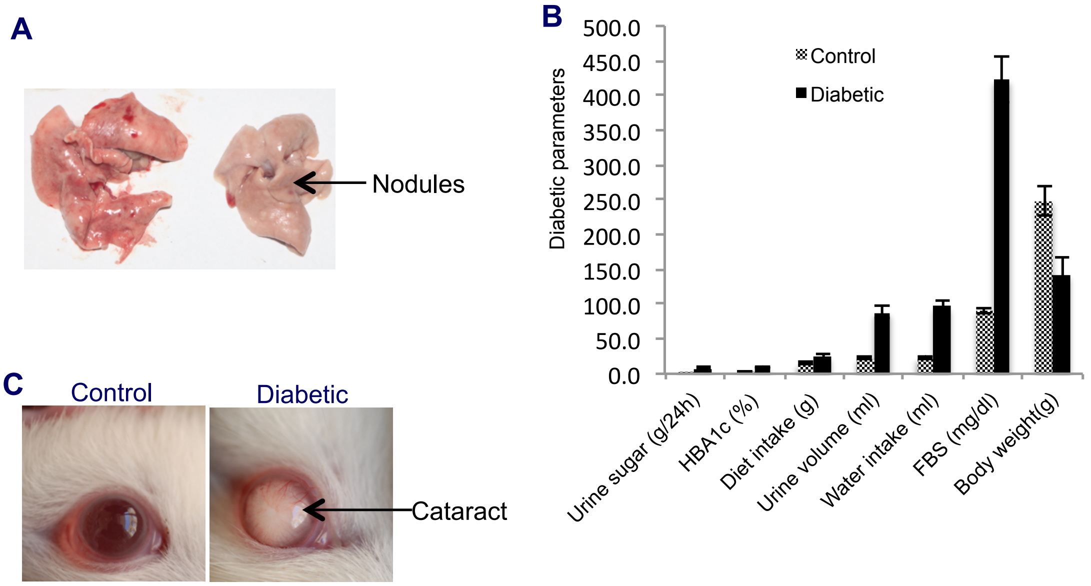

Fig. 1.

A. Diabetic parameters in STZ-injected rats. A total of 45 rats were injected with 45 mg/kg body weight STZ by the I/P route. At 12 wk postinjection, the animals were evaluated for diabetic markers, such as fasting blood sugar, HbA1c levels, diet intake, urine volume, water intake, fasting blood glucose levels, and body weight.

B. Cataract development in diabetic animals. A representative picture of a diabetic animal showing the cataract.

C. A representative picture showing the morphology of the lungs dissected from control and diabetic animals. The arrow mark shows the presence of nodules on the diabetic lung.

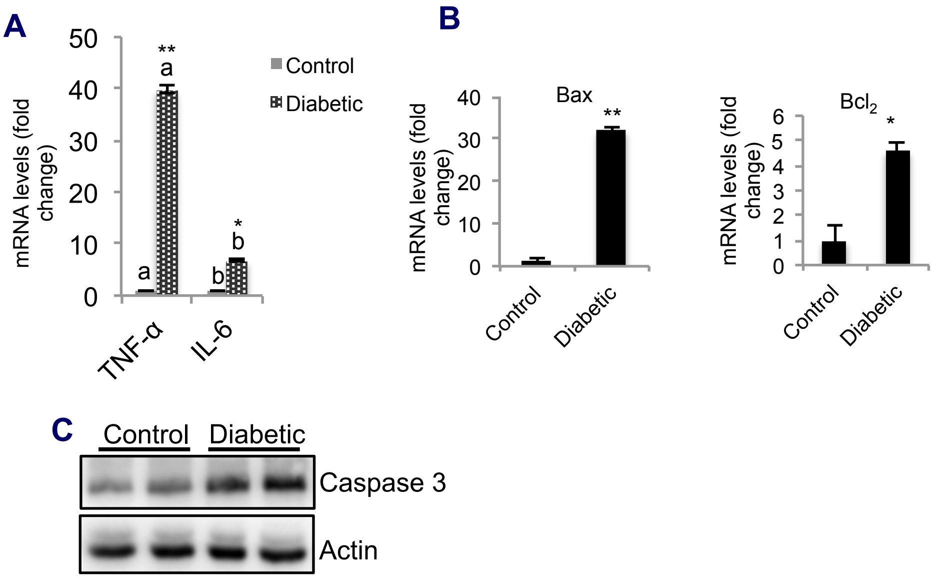

Fig. 2.

A. mRNA expression levels of the TNF-α and IL-6 genes by real-time PCR. Error bars represent the standard error of the mean of triplicate values. Compared to the control lung, the diabetic lung showed a significant upregulation of the TNF-α and IL-6 genes. The letters 'a' and 'b' on the bar graph indicate significant differences at $^*p<0.05$ or $^{**}p<0.01$; n=3.

B. mRNA expression levels of the Bax and Bcl2 genes by real-time PCR, as described above. Error bars represent the standard deviation of the mean of triplicate values. Compared to the control lung, the diabetic lung showed a significant upregulation of Bax and Bcl2 gene levels. Bar diagrams represent the mean±SE; n=3; $^*p<0.05$ and $^{**}p<0.01$.

C. Detection of caspase-3 expression in diabetic lungs by western blot analysis. Compared to the control lungs, the diabetic lungs showed more intense bands.

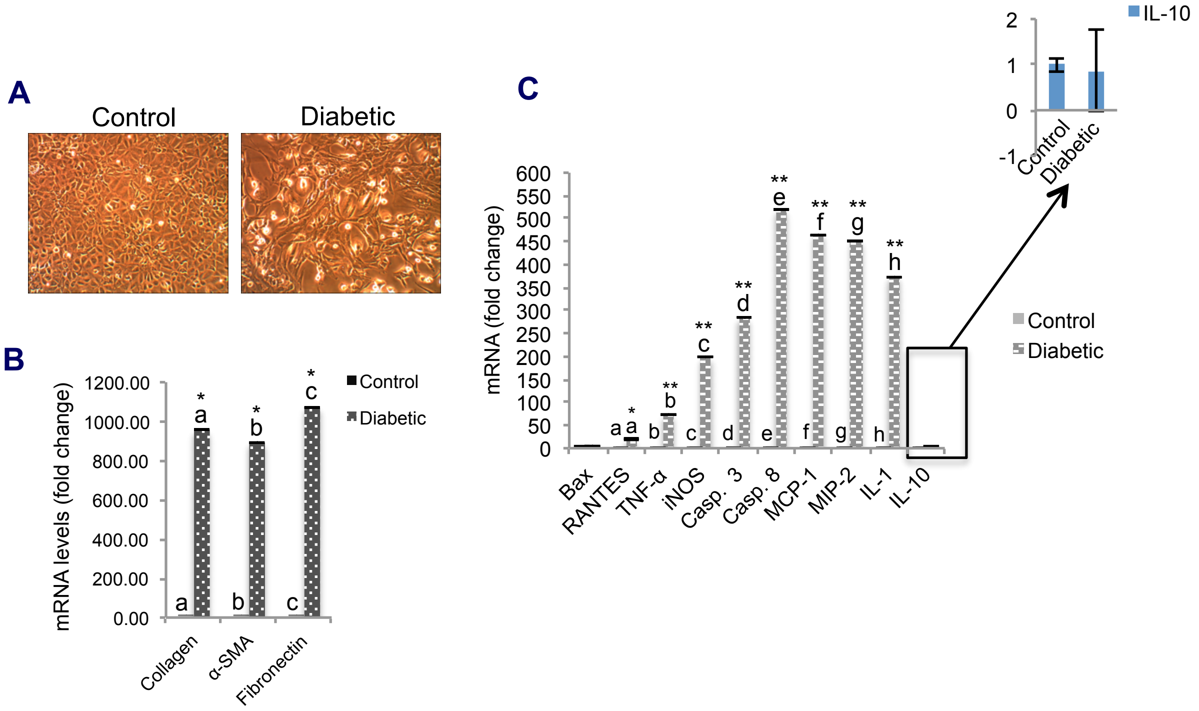

Fig. 3.

A. Representative images of control and diabetic cells isolated from control and diabetic lungs, respectively, 12 wk after diabetes induction. The diabetic cells showed a fibroblast phenotype, in contrast to the epithelial phenotype of control cells.

B. mRNA expression levels of fibroblast marker genes, such as collagen, α-SMA, and fibronectin, in control and diabetic cells by real-time PCR. Diabetic cells showed elevated expression levels of fibroblast maker genes. Bar diagrams represent the mean±SE; (*) and the letters ‘a’, ‘b’ and ‘c’ indicate significant differences at $p<0.05$; n=3.

A. mRNA expression levels of inflammatory and apoptotic genes in cultured cells by real-time PCR. Compared to the respective controls, diabetic cells showed a significant upregulation of the expression levels of inflammatory and fibrotic genes. However, the levels of the anti-inflammatory gene IL-10 (indicated by the arrow) were slightly decreased in diabetic cells compared to those in the control cells. Bar

graphs indicate the mean±SE; the letters ‘a’, ‘b’, ‘c’, ‘d’, ‘e’, ‘f’, ‘g’, and ‘h’ on the bar graph indicate significant differences at *p<0.05 and **p<0.01; (n=3).

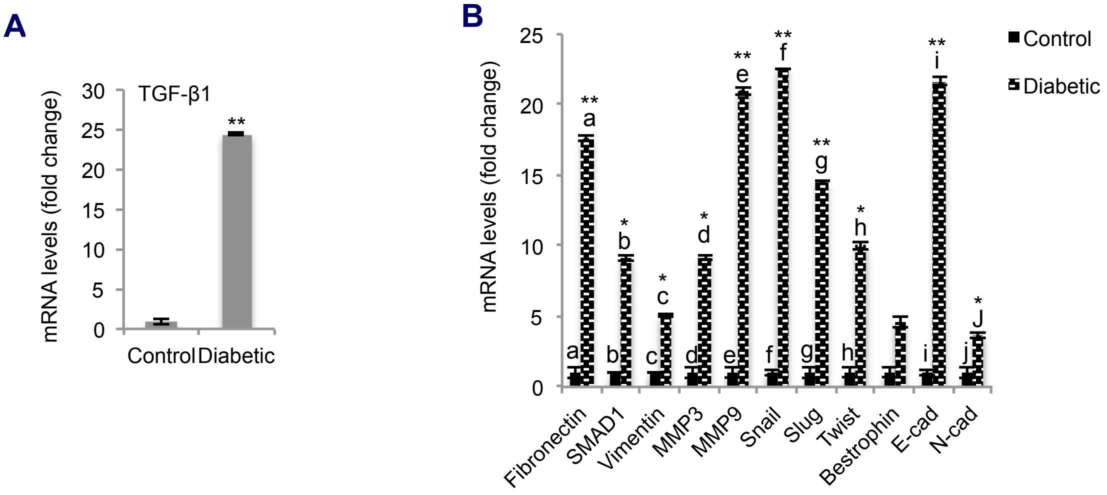

Fig. 4.

A. mRNA expression levels of the TGF-β1 gene by real-time PCR. Compared to the control lung, the diabetic lung showed a significant upregulation of TGF-β1 (24-fold) levels. Bar diagrams represent the mean±SE; **p<0.05; n=6.

B. mRNA expression levels of EMT marker genes by real-time PCR. Error bars represent the standard error of the mean of triplicate values. Compared to the control lung, the diabetic lung showed a significant upregulation of EMT marker gene levels. Note that E-cadherin levels were also significantly upregulated in the diabetic lung compared to those in the control lung. Bar diagrams represent the mean±SE; the letters ‘a’, ‘b’, ‘c’, ‘d’, ‘e’, ‘f’, ‘g’, ‘h’, ‘I’, and ‘j’ on the bar graph indicate significant differences at *p<0.05 and **p<0.01; n=3.

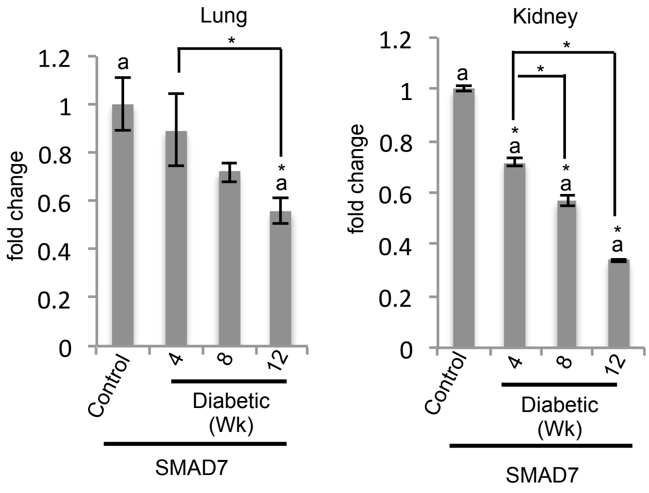

Fig. 5.

A. Densitometry analysis of SMAD7 in the diabetic lung and kidney. The diabetic lung and kidney were isolated from the same animal 4, 8, and 12 wk after diabetes induction and compared to control lung and kidney from the same animal 12 wk after buffer injection. The diabetic lungs showed no significant changes in the levels of SMAD7 until 8 wk, but those levels decreased significantly 12 wk after diabetes induction. In contrast, the diabetic kidney showed a significant decrease in SMAD7 after 4 wk, indicating that SMAD7 levels may play a role in delaying the effects of diabetes on the lung during the initial stages of diabetes induction. Bar diagrams represent the mean±SE; n=3; * $p<0.05$.

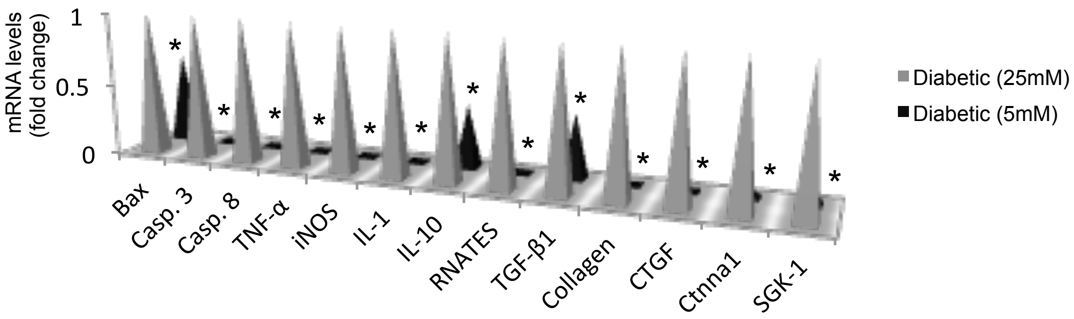

Fig. 6.

mRNA expression levels of inflammatory, apoptotic and fibrotic marker genes by real-time PCR. Bar graph represents the mean±SE; the letter 'a' denotes significant differences at *$p<0.05$; n=3. Compared to diabetic cells cultured in 25 mM glucose, glucose-restricted cells had downregulated levels of inflammatory, apoptotic and fibrotic genes.
